# Genetics of male infertility: Indian scenario

**DOI:** 10.1186/1755-8166-7-S1-I19

**Published:** 2014-01-21

**Authors:** K Thangaraj

**Affiliations:** 1 CSIR-Centre for Cellular and Molecular Biology, Hyderabad, India

## 

According to the recent epidemiological studies, nearly one out of every 10 couples face a problem in conceiving a child. Impaired fertility of male partner is causative in approximately 50% of all couples unable to conceive spontaneously. We have been studying the genetic factors associated with male infertility among Indian population. We have earlier shown that about 8.5% infertility among Indian men is due to the Y chromosome microdeletions. Further analysis of several autosomal (*NR5A1*, *KLK3*, *CETN1*, *DEFB126*, *CAMK4*, *UBE2B*, *TNP1* &*2* and *PRM1*, *2* &*3*); Y chromosomal (*DAZ* deletions, *TSPY1* copy number) and mitochondrial genes accounted for additional 19.5% of the genetic factors responsible for male infertility. However, etiology of a large proportion (72%) of infertile men still remains unknown. Gene expression studies from our lab using microarray approach, showed several genes are many folds down regulated in the testicular tissue of infertile men, compared to the fertile men. Targeted resequencing of these differentially expressed genes and functional characterization of the observed mutations is in progress. In addition, we have recently initiated exome sequencing of idiopathic male infertile samples to identify additional genetic factors responsible for male infertility. The results of the findings would be discussed at the time of presentation.

